# BSD: A Bayesian Framework for Parametric Models of Neural Spectra

**DOI:** 10.1111/ejn.70149

**Published:** 2025-05-26

**Authors:** Johan Medrano, Nicholas A. Alexander, Robert A. Seymour, Peter Zeidman

**Affiliations:** ^1^ Department of Imaging Neuroscience, Functional Imaging Laboratory UCL Queen Square Institute of Neurology London UK

**Keywords:** Bayesian inference, EEG, group‐level analysis, MEG, neural oscillations, spectral analysis

## Abstract

The analysis of neural power spectra plays a crucial role in understanding brain function and dysfunction. While recent efforts have led to the development of methods for decomposing spectral data, challenges remain in performing statistical analysis and group‐level comparisons. Here, we introduce Bayesian spectral decomposition (BSD), a Bayesian framework for analysing neural spectral power. BSD allows for the specification, inversion, comparison and analysis of parametric models of neural spectra, addressing limitations of existing methods. We first establish the face validity of BSD on simulated data and show how it outperforms an established method [fit oscillations and one‐over‐*f* (FOOOF)] for peak detection on artificial spectral data. We then demonstrate the efficacy of BSD on a group‐level study of electroencephalography (EEG) spectra in 204 healthy subjects from the LEMON dataset. Our results not only highlight the effectiveness of BSD in model selection and parameter estimation but also illustrate how BSD enables straightforward group‐level regression of the effect of continuous covariates such as age. By using Bayesian inference techniques, BSD provides a robust framework for studying neural spectral data and their relationship to brain function and dysfunction.

## Graphical abstract

We introduce Bayesian spectral decomposition (BSD): By performing Bayesian inversion of parametric models of neural spectral power, BSD enables straightforward group‐level analysis of spectral content and robust detection of spectral peaks. We demonstrate how BSD reveals the ageing effect on the spectral content of resting state occipital EEG signals.

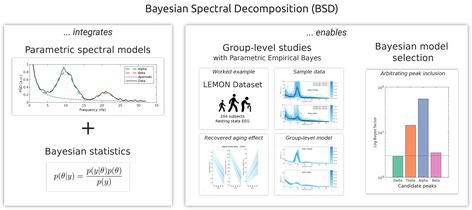

**Abbreviations**
AUC: area under curve
BSD: Bayesian spectral decomposition
DCM: Dynamic causal modelling
EEG: electroencephalographyFOOOF; fit oscillations and one‐over‐*f*
GLM: general linear model
PEB: parametric empirical Bayes
ROC: receiver operating characteristic
SNR: signal‐to‐noise ratio
SPM: statistical parametric mapping

## 1 Introduction

The spectral density of electrophysiological signals represents neurophysiological processes as a combination of rhythmic oscillations and aperiodic power with apparent scale‐free behaviour (Donoghue et al. 2020; He 2014). Rhythmic oscillations are assumed to play a fundamental role in neuronal computations (Buzsáki and Watson 2012), and as such, they are observed in electrophysiological measurements of most brain disorders and neuroimaging experiments (Buzsaki and Draguhn 2004). They have been hypothesised to enable communication between cortical populations (Fries 2005), thereby coordinating the emergence of centre manifolds that provide a stable scaffold on which cortical computations can take place (Huys et al. 2014). The remaining, aperiodic part of the spectrum, which exhibits a scale‐free 1*/f* spectral density, also reflects neurophysiology (He 2014; Allegrini et al. 2009). For instance, changes in the spectral aperiodic component related to healthy ageing are well known (Hill et al. 2022; Thuwal et al. 2021), as well as in disorders such as ADHD (Karalunas et al. 2022; Arnett et al. 2022). Both the periodic and aperiodic components are therefore relevant for clinical and cognitive neuroscience.

Recent efforts have been focused on finding systematic approaches for decomposing spectral neural data (Whitten et al. 2011; Wen and Liu 2016). One recent and popular method is fit oscillations and one‐over‐*f* (FOOOF) (Donoghue et al. 2020), also known as spectral parameterisation (SpecParam). FOOOF parameterises log power spectral densities as the sum of periodic and aperiodic components that are estimated from the data using an iterative procedure. Despite its relatively recent introduction, FOOOF has already been successfully applied in hundreds of different studies, either to isolate and analyse the rhythmic oscillations (Tröndle et al. 2023), the aperiodic component (Merkin et al. 2023), to remove the confounding effect of the aperiodic component for group studies (Oswal et al. 2021) or to improve the detection of transient oscillations (Seymour et al. 2022). This highlights the strong relevance of the spectral model offered by FOOOF for addressing research questions about brain function and disorder.

Despite being a successful tool for decomposing power spectra, FOOOF may benefit from several improvements. For instance, FOOOF relies on selecting several parameters that can influence the results, but there is no well‐established approach to select these parameters. In addition, there is no straightforward way to perform statistical analysis with FOOOF; in particular, there is no standardised approach for performing group‐level analysis. Some authors have used FOOOF outputs to perform statistical analysis (Oswal et al. 2021). However, these approaches fail to account for the uncertainty of parameters estimates: ideally, one would like to convey both estimated parameters and their confidence (precision) from the individual subjects to the group‐level.

Fortunately, these limitations can be addressed by moving to a Bayesian framework. Bayesian statistics have already proven to be useful in other neuroimaging applications (Friston, Glaser, et al. 2002; Friston, Penny, et al. 2002). A range of tools for model‐estimation, group‐level analysis and model selection have been devised and improved over the two last decades in neuroimaging (Zeidman et al. 2023). Specifically, we can leverage variational Bayesian inversion to estimate parameters (Friston et al. 2003, 2007), Bayesian model selection to choose the most adequate model (Kass and Raftery 1995; Stephan et al. 2009) and parametric empirical Bayes (PEB) to conduct between‐subject analysis while accounting for within‐subject variability (Friston et al. 2016; Zeidman et al. 2019). Overall, Bayesian methods enable standardised, integrated pipelines for performing statistical analysis of single‐subject and group‐subject data, which we can leverage for analysing neural spectra.

In this work, we introduce Bayesian spectral decomposition (BSD): a Bayesian framework for specifying, estimating, comparing and analysing parametric models of neural spectral power. This article is structured as follows. We first present the theoretical elements of BSD, focusing on introducing only the immediately relevant concepts from Bayesian inference. We then proceed with establishing its face validity on simulated data, before showcasing its application in a large‐scale group‐level study of the ageing effect on the EEG spectra of 204 healthy subjects from the LEMON dataset. Finally, we discuss the potential applications BSD enables and the future directions for its development.

## 2 Theory

### 2.1 Parametric Spectral Model

BSD models oscillatory and scale‐free patterns in the spectral density or log‐spectral density. The parametric spectral model given here focuses on modelling the amplitude spectral density but can be straightforwardly adapted to fit the log‐spectral density. The amplitude spectral density obtained as the square‐root of the power spectral density computed using any approach, for example, fast‐Fourier transform, Welch's method or using an autoregressive model. Prior to computing the spectral density, we scale the signal to a variance of 8. This optional step removes the need to scale model parameters to the scale of the signal, but can be omitted if the signal variance conveys meaningful information for the analysis.

The noise‐free spectral density *S*_*f*_ (*θ*), for frequencies *f* = (*f*_1_, …, *f*_*n*_) and some parameters *θ*, is modelled as the sum of an aperiodic component (*A*_*f*_) and *N* periodic components (*P*_*f,i*_):

(1) $$S_{f}\left(\theta \right)=A_{f}\left(\theta \right)+\sum_{i=1}^{N}P_{f,i}\left(\theta \right)$$

Each periodic component characterises a peak in the spectrum. Given that we are principally interested in the mean and width of a peak, a Gaussian model is suitable. Here, a single Gaussian peak has three parameters—a mean *m*_*i*_, a standard deviation *s*_*i*_ and a height *h*_*i*_, where *i* indexes the peak. It has the following form:

(2) $$P_{f,i}\left(\theta \right)=h_{i}\left(\theta \right)\exp\left(-\frac{1}{2}{\left(\frac{f-m_{i}\left(\theta \right)}{s_{i}\left(\theta \right)}\right)}^{2}\right)$$

The aperiodic component captures the power scaling law commonly observed in the spectrum of biophysical signals. It has the form:

(3) $$A_{f}\left(\theta \right)=\gamma \left(\theta \right)f^{\alpha \left(\theta \right)}$$

where *γ* is the broadband amplitude and *α* is the (negative) scaling exponent. This model can be used to fit the log‐spectral density by replacing the aperiodic component (*A*_*f*_ (*θ*)) in (1) with its logarithm (log *A*_*f*_ (*θ*)). The noise‐free parametric spectral model, composed of the periodic and aperiodic components, forms the basis of the spectral likelihood.

### 2.2 Spectral Likelihood

In practice, the observed spectrum *Y*_*f*_ is corrupted by various sources of noise and unmodelled effects. We account for these by adding a noise term *ε*_*f*_ to the spectral density:

(4) $$Y_{f}=S_{f}\left(\theta \right)+\varepsilon_{f}$$

*ε*_*f*_ is assumed to follow a multivariate Gaussian distribution with zero mean and covariance *C*_*f*_ (*η*), where *η* is a precision parameter determining the scale of the noise:

(5) $$\varepsilon_{f}∼N\left(0,C_{f}\left(\eta \right)\right)$$

This noise is generic and does not impose dependence between frequencies. One can consider the Gaussian form of noise as an approximation for noncentral chi‐distributed spectral noise that would arise from additive Gaussian noise in the temporal domain. The covariance matrix *C*_*f*_ is defined as follows:

(6) $$C_{f}\left(\eta \right)=\eta^{-1}R_{f}$$

where *R*_*f*_ is the correlation matrix computed from the cross‐spectral density of the signal, following (Camba‐Mendez and Kapetanios 2005; Friston et al. 2012).

Together, Equations (4) and (5) prescribe a spectral likelihood model that gives the probability of the spectrum.

*Y*_*f*_ from parameters $\theta ≔\left\{\theta ,\eta \right\}$:

(7) $$Y_{f}|\theta ∼N\left(S_{f}\left(X\theta \right),C_{f}\left(\eta \right)\right)$$

For most parameters, some values are more probable that others. This can be incorporated in the model through parameter priors.

### 2.3 Priors and Constraints

Parameter priors reflect any knowledge we have about parameters before observing the data, for example, preferred values and constraints. The choice of priors is delicate as it can make the model more complex, computationally intensive or numerically unstable. To address these issues, we restrict priors to the well‐behaved multivariate Gaussian family:

(8) $$\theta ∼N\left(\mu_{\theta },\Sigma_{\theta }\right)$$

(9) $$\eta ∼N\left(\mu_{\eta },\Sigma_{\eta }\right)$$

(10) $$\;p\left(\theta \right)=p\left(\theta \right)p\left(\eta \right)$$

The last equation reflects the standard assumption of independence between parameters of interest and precision parameters that govern noise. In brief, using Gaussian priors amounts to saying that we are only interested in the mean and (co‐)variance of our model parameters.

Naturally, some model parameters do not fit the Gaussian assumptions, as they must respect some constraints—for instance, a peak height must be positive. For this reason, model quantities in Equations (2) and (3) are mapped from parameters through link functions. Table 1 summarises link functions and prior expectations and covariances used in BSD. Note that the prior distributions provided here are deliberately imprecise and therefore quite generic, but could be refined by population information or tailored to a specific study. The most common transform is the exponential transform, which gives a model parameter a log‐normal distribution and ensures its positivity.

**TABLE 1:** Parameters of interest, their link function and default priors.

Parameter	Symbol	Link function	Priors
Aperiodic exponent	*α*	*α*(*θ*) = *−*exp (*θ*_*α*_)	*θ*_*α*_ ∼ N(0*,* 3)
Aperiodic power	*β*	*β*(*θ*) = exp (*θ*_*β*_)	*θ*_*β*_ ∼ N(0*,* 3)
Peak power	*h*_*i*_	*h*_*i*_(*θ*) = exp (*θ*_*hi*_)	*θ*_*hi*_ ∼ N(0*,* 2)
Peak frequency	*m*_*i*_	*m*_*i*_(*θ*) = *L*_*i*_ + *σ* (*θ*_*mi*_) (*U*_*i*_ *− L*_*i*_)	*θ*_*mi*_ ∼ N(0*,* 2)
Peak width	*s*_*i*_	*s*_*i*_(*θ*) = exp (*θ*_*si*_)	*θ*_*si*_ ∼ N(0*,* 2)

*Note:* The function *σ* appearing in the link function for the peak frequency is the hyperbolic arc‐tangent.

**FIGURE 1:**
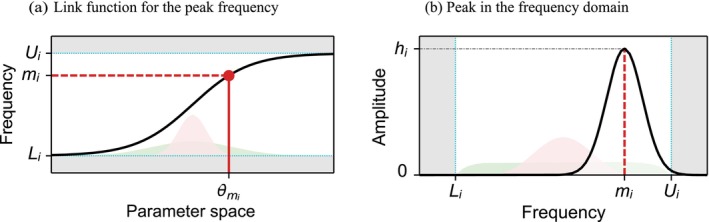
Parameterisation of the peak frequency *m*_*i*_. In panel (a), the unconstrained parameter *θ*_*mi*_ is shown on the horizontal axis. Before it is used within the model, it is transformed to the peak frequency *m*_*i*_ on the vertical axis (black curved line). Importantly, we see that the peak frequency is softly clipped between the band parameters *L*_*i*_ and *U*_*i*_ (dotted cyan lines). The resulting value of *m*_*i*_ (dashed red line) parameterises the mode of the Gaussian peak and is constrained between the frequency band limits, as shown in panel (b). Green and pink shaded areas, on the *x*‐axis, display how Gaussian distributions over *θ*_*mi*_ with different variances (panel (a)) induces different distributions over the frequency band (right (b)). By default, we set priors as displayed in the green shaded area.

Importantly, we have used a hyperbolic arc‐tangent function to map each periodic component to disjoint sections of the frequency domain (referred to as *frequency bands* in the following) (Figure 1). Inherently, this means that BSD is modelling a unique peak per frequency band. This restriction prevents the recruitment of several periodic components to fit a single peak. In addition, it ensures that the peaks estimated for different subjects are all in comparable frequency ranges, such that it makes sense to analyse them together. In other words, restricting the model to one peak per frequency band makes the model more interpretable and simpler to compare across subjects. Note that frequency bands should be specified broadly enough to accommodate the full range of peak displacement across subjects.

### 2.4 Model Estimation

As BSD is a Bayesian method, model estimation should produce two quantities: the *posterior distribution—*that is, the probability distribution of the parameters given the observed spectrum, *p*(***θ**|Y*_*f*_)*—*and the *log marginal evidence—*that is, the log‐probability of the observed spectrum under the model, ln *p* (*Y*_*f*_). As this problem is generally intractable, BSD relies on variational inference. Variational inference introduces an *approximate posterior*, *q*_*ϕ*_(***θ***), and an approximate log marginal evidence: the *free‐energy*, *F* (details about the algorithm, with accompanying code, can be found in Zeidman et al. 2023). At convergence, we have

(11) $$q_{\phi }\left(\theta \right)\approx p\left(\theta |Y_{f}\right)$$

(12) $$F\approx \ln\;p\;\left(Y_{f}\right)$$

which provides an approximate solution to the Bayesian inference problem. The probabilistic estimates of the parameters, *q*_*ϕ*_(***θ***), are then collated across subjects to enable group‐level analysis.

### 2.5 Group‐Level Analysis

Once the spectral model of each subject has been estimated, one can conduct group‐level analysis on model parameters. The motivation is to study and compare spectral effects at the group level, for instance, to quantify changes in the location or width of a peak between pathological and healthy subjects. For this, BSD leverages the PEB framework (Friston et al. 2016; Zeidman et al. 2019). In brief, PEB allows one to rapidly perform Bayesian second‐level analysis using a general linear model (GLM). Consider a group of *N* individuals, where subject *k* has spectral data *Y*_*k*_. PEB models the data as

(13) $$Y_{k}=S_{f}\left(\theta_{k}^{\left(1\right)}\right)+\varepsilon_{k}^{\left(1\right)}$$

(14) $$\theta^{\left(1\right)}=X^{\left(2\right)}\theta^{\left(2\right)}+\varepsilon^{\left(2\right)}$$

The first line is the spectral likelihood seen before, which accounts for within‐subject effects: each subject is modelled with their own parameters $\theta_{k}^{\left(1\right)}$ and observation noise $\varepsilon_{k}^{\left(1\right)}$. Group‐level effects are modelled by a GLM with design matrix **X** ^(2)^ mapping parameters *θ* ^(2)^ to subject‐level model parameters. Both the priors on the parameters governing the second‐level noise and the parameter priors are assumed to follow multivariate normal distributions. In practice, BSD uses the default priors provided in PEB routines in the Statistical Parametric Mapping (SPM) software, thus only the design matrix needs to be specified to perform a second‐level analysis (Friston et al. 2016; Zeidman et al. 2019, 2023). Group‐level analysis with PEB is conducted in three steps. First, one inverts each subject's model using gradient ascent on the variational free‐energy to obtain the posterior distribution of each subject's parameters. Second, one specifies the second‐level design matrix. Third, second‐level model parameters are optimised to maximise the free‐energy for the entire hierarchical model (accounting for both within‐ and between‐subjects effects) (Friston et al. 2016). Note that this approach assumes that subjects share the same spectral model and that different parameterisation of that model give rise to the observed spectral differences. This assumption is not constraining: if some subjects lack one of the spectral peaks, the model will simply estimate a zero height for that peak. Naturally, if that effect is dominant across the group, one would expect a simpler model without that peak to be favoured—this conservative behaviour is the essence of Bayesian model comparison, and is sometimes referred to as the ‘principle of parsimony’.

### 2.6 Bayesian Model Comparison

Bayesian model comparison is the Bayesian approach to making modelling decisions and mitigating between alternative explanations for the data (Stephan et al. 2009; Friston et al. 2016; Kass and Raftery 1995). In BSD, it enables comparing different spectral models and making a principled selection, for example, the number of periodic components to include in the model or their frequency band.

Consider two competing hypotheses *H*_1_ and *H*_2_, for example, whether the data contains an alpha peak. These translate into two models *m*_1_ and *m*_2_ with different spectral likelihood and priors. Having observed spectral data *Y*_*f*_, we want to assess whether one hypothesis is more probable than the other. Formally, *H*_1_ being *β*_12_ times more probable than *H*_2_ after observing *Y*_*f*_, translates as *p*(*m*_1_ *|Y*_*f*_) = *β*_12_ *p*(*m*_2_ *|Y*_*f*_). Assuming that both hypotheses are equiprobable a priori, that is, *p*(*m*_1_) = *p*(*m*_2_), Bayes rule gives

(15) $$\beta_{12}=\frac{p\left(m_{1}|Y_{f}\right)}{p\left(m_{2}|Y_{f}\right)}=\frac{p\left(Y_{f}|m_{1}\right)p\left(m_{1}\right)}{p\left(Y_{f}|m_{2}\right)p\left(m_{2}\right)}=\frac{p\left(Y_{f}|m_{1}\right)}{p\left(Y_{f}|m_{2}\right)}$$

The factor *β*_12_ is called the Bayes factor (Kass and Raftery 1995). From Equation (15), the Bayes factor between two equiprobable hypotheses is the ratio of the model evidence of their corresponding probabilistic models. Or more simply, selecting between hypotheses amounts to constructing their probabilistic models and comparing their ability to predict the data. A commonly accepted interpretation of the values of Bayes factors are shown in Table 2.

**TABLE 2:** Common interpretation of evidence strength from Bayes factors, adapted from Kass and Raftery (1995).

ln *β*_12_	*β*12	Evidence towards *m*_1_
*<* 0	*<* 1	Negative (towards *m*_2_)
0 to 1	1 to 3	Weak
1 to 3	3 to 20	Positive
3 to 5	20 to 150	Strong
*>* 5	*>* 150	Very strong

In variational inference, the marginal evidence is approximated by the free‐energy (Equation 12). Accordingly, we can estimate the log Bayes factor between the two models as:

(16) $$\ln\;\beta_{12}\approx F_{m_{1}}-F_{m_{2}}$$

where *F*_*mi*_ is the free‐energy of the model after convergence. Thus, model comparison is conducted by first estimating each model and then comparing their free‐energy.

Notably, Bayes factors are likelihood ratios—that is, ratios of data likelihood under competing models. By the Neyman–Pearson lemma, mitigating between hypothesis using a likelihood ratio is the most powerful (i.e., sensitive) test across all possible levels of specificity. In other words, using Bayes factors as a decision criterion for model comparison maximises the receiver operating characteristic (ROC)‐area under curve (AUC) curve. For BSD, this ensures that Bayesian model comparison is the most powerful way to detect periodic components. In practice, this implies that all modelling questions arising while creating a model (e.g., should the model include a peak in the alpha range, or should the theta and alpha band be modelled separately) can be safely and straightforwardly answered using Bayesian model comparison.

### 2.7 Additions to the Spectral Model

The proposed generative model for the spectrum can be easily expanded without changing the procedure detailed above. This can be done by updating the likelihood model and specifying priors for any additional parameter. Extensions include modelling the effect of filters, condition‐specific effects, and source level data.

#### 2.7.1 Filter Modelling

In practice, the spectral data has undergone several filtering steps before being analysed. In BSD, the knowledge of the filtering history and each filter's parameter can be leveraged to account for information lost in filtering. Having applied *K* filters, where filter *k* has transfer function *H*_*k*_, the spectral model becomes

(17) $$Y_{f}=\sum_{k=1}^{K}\left|H_{k}\left(f\right)\right|S_{f}\left(\theta \right)+\varepsilon_{f}$$

This is especially important as the covariance of *ε*_*f*_ is estimated from the data. Thus, filtered regions will invariably have a low covariance. The model might thus become over‐precise in filtered regions. Including the filtering effect on the spectral amplitude acknowledges this reduction in variance, and reduces the risk of overfitting data in filtered regions.

#### 2.7.2 Condition‐Specific Effects

In most empirical studies, the interesting results do not lie in the absolute characterisation of data features but rather in quantifying their relative change between different experimental conditions—for instance, quantifying how much the peak height varies between rest and task. BSD accounts for condition‐specific effects at the individual level using a linear mapping from conditions to parameters. Assuming several conditions, where *Y*_*f,c*_ is the spectral data for condition *c*, Equation (4) is adapted as

(18) $$Y_{f,c}|\theta ∼N\left(S_{f}\left(X_{c}\theta \right),C_{f}\left(\eta \right)\right)$$

*X*_*c*_ plays the role of a design matrix and maps a linear combination of parameters to each condition.

#### 2.7.3 Source and Sensor Space

BSD allows one to analyse the spectral data in the sensor or source domain. In the source domain, both the forward model and prior on source location must be specified. Then, a current source density approach is used to project the modelled spectrum at source level onto the measured spectrum at sensor level. Importantly, the source locations are formulated as priors; thus, the location of precise sources can be refined from the data. In both source and sensor space modes, the frequency and width of each periodic components can either be shared between all channels or estimated independently for each channel. In addition, the formulation allows one to estimate at the same time a common aperiodic component and several aperiodic components for each individual source or sensor. This can help distinguish region‐specific aperiodic components from the global background activity.

## 3 Results

### 3.1 Face Validity

#### 3.1.1 Simulation Procedure

**FIGURE 2:**
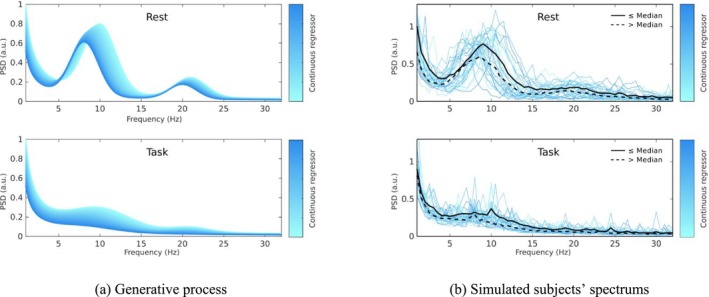
Spectrum and samples of the simulated example. The left panels show the generative process, that is, the model used to generate the data, in the rest and task conditions. The spectrum in each condition is influenced by a continuous regressor, indicated by the shading of the line, which can be seen as a subject trait such as age. The right plot shows the sampled spectra in both conditions for the 32 subjects. The plain black line shows the spectral average for subjects with a continuous regressor value below the population median, and the dashed line corresponds to the average for subjects above the median.

We first establish the face validity of BSD on simulated data. We construct an example that features two conditions, labelled ‘rest’ and ‘task’. We a spectral model with a large peak in the alpha band (defined as 8 to 12 Hz) and a smaller peak in the beta band (defined as 12 to 30 Hz) in the rest condition. Akin to eyes close/eyes open experiments, the effect of the task condition is to severely attenuate both peaks. In addition, we introduce a continuous covariate, for example, representing age, that differs for each subject and modulates their spectral parameters. In Figure 2, we display the generated spectrum in both conditions as a function of the continuous regressor.

Having established the generative process, we sample the spectrum of 32 artificial subjects. To sample a spectrum from a subject, we first sample a random value of the continuous covariate, compute the corresponding spectral parameters, and add centred Gaussian noise to these parameters. The noise variance is set to achieve 15% of the mean of the parameters of interests (i.e., after applying the link functions from Table 1). We then compute the spectrum for frequency between 1 and 32 Hz using Equation (1) and add observation noise. This gives 64 spectra, shown in Figure 2, which play the role of our experimental observations. Note that this generation procedure does not necessitate one to create the data in the time domain.

#### 3.1.2 Model Selection

**FIGURE 3:**
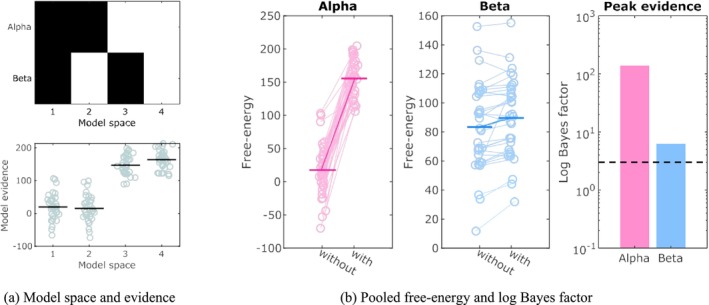
Model comparison and frequency band selection. The model space is created by having alpha and beta peaks either absent or present, as shown on the upper left panel (black = absent, white = present). Models 3 and 4 have an alpha peak and models 2 and 4 have a beta peak. Model evidence, shown on lower left plot, are obtained by inverting each model for each subject. In this panel, each marker represents the log‐evidence of a subject, and the horizontal bars show the group average. The two plots on the left of panel (b) are obtained by averaging the free‐energy of models without and with a particular peak. The average free‐energy increase caused by introducing the alpha or beta peak is the log‐Bayes factor, shown on the right plot of panel (b). The dotted horizontal lines highlight a log Bayes factor of 3, which corresponds 20 times probability factor and is commonly considered as strong evidence (see Table 2).

We first need to select a model for our data. The model space consists of all models to be compared, where each model is defined by a particular set of frequency bands. Without any prior knowledge on the frequency content of the signal, one can populate the model space by exploring all possible combinations of a set of frequency bands of interest. In practice, one can refine the model space by introducing some hypotheses about what periodic peaks might be present. In this example, a visual inspection of the individual spectra and their median split suggests the presence of an alpha peak and to a lesser extent, that of a beta peak. The next step is to confirm the presence of each peak using Bayesian model comparison. In this example, there are two possible peaks and thus four candidate models: no peaks, beta peak only, alpha peak only or both peaks. This forms the model space, which is illustrated in Figure 3.

We next invert each model in our model space on data from each artificial subject. After optimisation, we obtain an estimate of the marginal evidence (free energy) under each model and each subject (Figure 3a). We observe that models that include an alpha peak have much higher evidence than models without it (Figure 3a). For the beta peak, the difference is more subtle. We average the evidence with and without an alpha peak, and that with and without a beta peak. Results are shown in Figure 3b. We see that the presence of both alpha and beta peak increases the free‐energy. To confirm the significance of these findings, we compute the free‐energy difference, which is a proxy for the log Bayes factor (Equation 16). We observe a log Bayes factor of more than 100 for the alpha peak, and more than 6 for the beta peak. Thus, according to the interpretation of log Bayes factors (2), we can conclude that we have found very strong evidence for the presence of an alpha peak, and strong evidence for the beta peak. The best model for our data is model 4, which includes both alpha and beta peaks. Indeed, this corresponds to the model used to generate the data, and highlights the adequacy of Bayesian model comparison to uncover the right model.

#### 3.1.3 Effect of the Continuous Regressor

**FIGURE 4:**
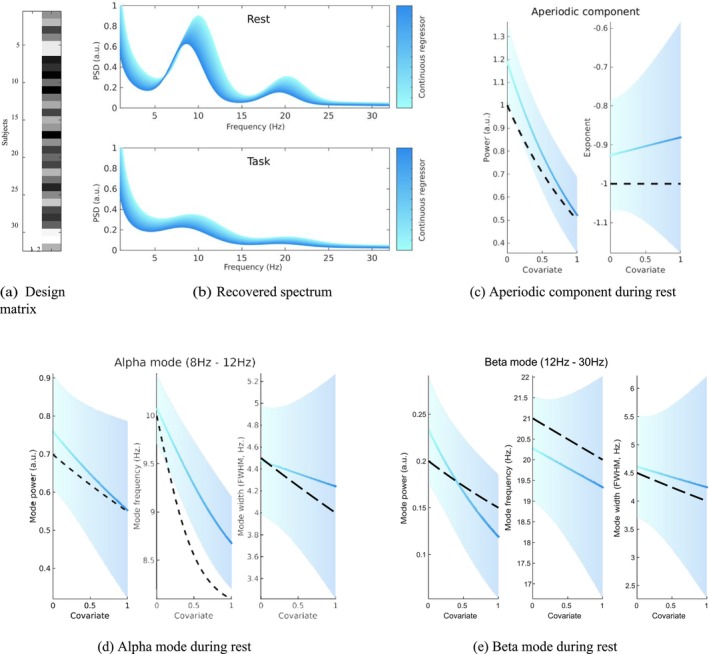
(a) Design matrix used for second‐level analysis, with an intercept as first column and the continuous regressor as second column. (b) Recovered spectral density, showing both an effect of the continuous regressor and an effect of the task condition. (c) Effect of the continuous regressor on the power (left) and exponent (right) of the aperiodic component. The dashed black line indicates the value that was used to generate the data. The blue line indicates regressed mean of the effect. The shaded area surrounding the mean indicates the 90% credible interval derived from the variance of the posterior distributions. (d) Effect of the continuous regressor on the power (left), frequency (middle) and width (right) of the alpha mode. (e) Effect of the continuous regressor on the power (left), frequency (middle) and width (right) of the beta mode.

So far, we have identified that the model with both alpha and beta peaks is the best model for this data. For this, we have inverted a model for each subject, and obtained both the model evidence and the posterior parameter distribution. In addition, for each subject, we have the value of the continuous covariate, for example, age. We now want to test whether the within‐subject parameters are associated with the continuous covariate at the group level. This translates into writing a general linear model that explains the estimated posterior distribution of the subjects' parameters as a function of the continuous covariate, plus an intercept capturing the average parameter value for the population. The design matrix corresponding to this linear model is displayed in Figure 4a.

We use PEB to estimate the model coefficients. After applying PEB, for each of the 8 parameters of our model (2 for the aperiodic component, and 2 *×* 3 for the periodic component), we have the estimated parameter average across the population, and its association with the covariate. These two parameters configure the intercept and slope of a linear mapping of the parameter against the covariate. As we are working in a Bayesian framework, we also have credible intervals for these two effects, and thus for the entire curve. In other words, we can recover the mean and credible interval for the power and exponent of the aperiodic component (Figure 4c) and the power, frequency and width of the alpha (Figure 4d) and beta (Figure 4e) modes. We can see that for most quantities, the true effect falls within the credible interval of our model. This is not the case for the frequency decrease of the alpha mode frequency, where the estimated effect is overconfident. Overconfidence in variational inference is a known effect that has been reported elsewhere (see Daunizeau et al. 2009; Friston, Penny, et al. 2002). Because we have the full model of how parameters change with the covariate, we are now in position to interpolate the spectrum over the entire covariate range of the population. This gives the plot Figure 4c, which we can compare with that of the generative process (Figure 2). We see that BSD recovers all the effects of interest. Note that this experiment does not address the sensitivity and specificity to the presence of a peak, which is the focus of the next section.

### 3.2 Sensitivity and Specificity: Comparison With FOOOF

In this section, we evaluate the sensitivity and specificity of BSD when used to assess the presence of a peak in a spectrum. We also compare both metrics with an established method, FOOOF. We are interested in observing how the statistical power of both methods changes with the peak height, the peak location, and the amount of observation noise.

We generate spectra with a single peak within a frequency band. We investigate the following frequency bands: delta (1 to 4 Hz), theta (4 to 8 Hz), alpha (8 to 12 Hz), beta (12 to 30 Hz) and gamma (30 to 64 Hz). For each band, we generate 1024 random spectra over a frequency grid from 1 to 64 Hz with 0.5 Hz resolution. Each spectrum has a random peak location and width, and random aperiodic parameters. The peak height is set to a fixed Signal‐to‐Noise Ratio (SNR), defined as a fixed factor of the sum of the aperiodic component and variance of the output noise. Similarly, the variance of the output noise is set to a fixed fraction of the scale of the aperiodic component.

For each spectrum, we invert two BSD models: a null model, without any peak, and an informed model, with a peak in the correct frequency band. After inversion, we compute the log Bayes factor between the two models and use it as a criterion to determine whether a peak is present or absent. Separately, we estimate a FOOOF model using the specparam Python package with default parameters (from specparam V1.1.0). We use the height of the highest peak in the correct frequency band as a criterion for FOOOF. We specify a height of 0 if FOOOF did not find any peak in the correct frequency band, which happened only rarely. This ensures that both FOOOF and BSD are informed by the frequency band.

**FIGURE 5:**
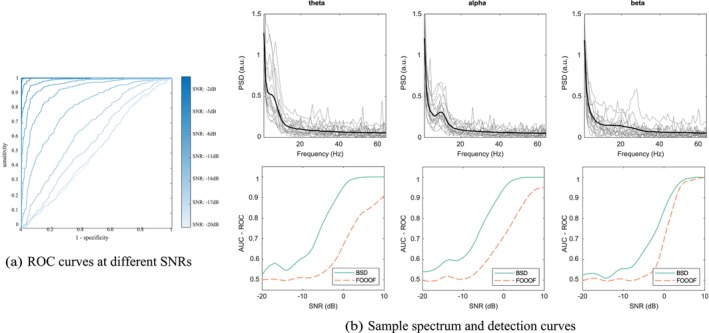
(a) ROC curves for detecting a beta peak under different levels of SNR. (b) Sample spectrum and detection curves for the theta (left), alpha (middle), and beta (right) frequency bands. The first row shows sample power spectral density for a log precision of the noise of 4 and a SNR of 1 dB. The overlaid black curve shows the sample spectral average. The second row shows the AUC‐ROC, that is, the detection score, as a function of the peak signal‐to‐noise ratio in decibels for both BSD (in plain green) and FOOOF (in dashed orange). For FOOOF, the default parameters of the official Python implementation (specparam) have been used.

Having defined a criterion for each model, we compute its receiver operating characteristics. This is done by plotting the specificity against the sensitivity of the model, under all possible values of decision threshold. In other words, we set a numerical threshold to compare the criterion against and determine the presence of a peak, compute the specificity and sensitivity of the test, and repeat these steps for all possible threshold values. Sample ROC curves are shown in Figure 5. The ROC can be summarised by the AUC. We compute the ROC‐AUC for BSD and FOOOF. We then plot the ROC‐AUC as a function of the peak SNR. Key results are displayed in Figure 5, and complete results are attached in the Supporting Information.

For all frequency bands and SNRs, we see that both BSD and FOOOF manage to uncover the true peak when its height is large enough. Interestingly, for all plots, we see that BSD is much better than FOOOF in detecting peaks of intermediate height, and has a higher AUC overall. This is a nice illustration of Neyman–Pearson lemma, which states that likelihood ratios have the maximum ROC‐AUC.

This result may be contrasted against very small peaks with SNR under −10 dB. There, BSD can have a ROC‐AUC comparable or under that of FOOOF and even under 0.5 (see Supporting Information). Naively, the latter case means that BSD performs ‘worse’ than deciding randomly whether a peak is present. This reflects a more subtle and important effect inherited from the use of variational inference. Indeed, we use the free‐energy to compute the log Bayes factor. By construction, the free‐energy expresses a trade‐off between accuracy and complexity. When a peak is too small, models with and without that peak have the same accuracy but introducing a peak increases the model complexity. Thus, the approximate log Bayes factor points us towards the simplest explanation for the data, that is, the model without a peak, which does not correspond to the generative process and thus counts as a false negative. Hence, this result is observed only for very low SNRs (less than −10 dB, i.e., a noise amplitude 10 times larger than the signal amplitude) and reflects a conservative aspect of BSD, which favours a simpler explanation (i.e., not reporting a peak) when the data does not justify a more complex one (i.e., a peak that can be mistaken for noise).

### 3.3 Worked Example: Applying BSD to the LEMON Dataset

#### 3.3.1 Data Presentation

In this section, we show how BSD can be deployed to analyse the frequency content of real EEG data and the variation of spectral parameters with age. For this, we use the LEMON dataset (Babayan et al. 2019; Mendes et al. 2019), which features 202 subjects and includes experimental EEG data with eyes closed and eyes open conditions. In addition, the age range of each subject is reported, with a resolution of 5 years.

We aim at building a simple model of the spectral density at channel Oz in both conditions, expressed using a commonality and condition‐specific parameterisation (Equation 18). In addition, we leverage the statistical power of the sample size to estimate a linear model of parameters as a function of age, across the entire group.

#### 3.3.2 Model Selection

**FIGURE 6:**
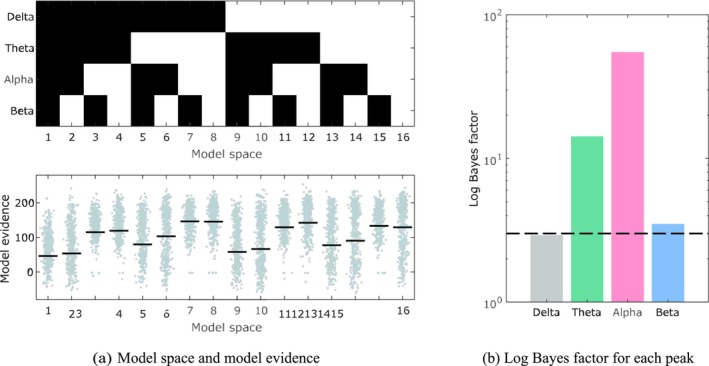
Bayesian model comparison results for the LEMON dataset. (a) The upper most plot indicates the absence (black) or presence (white) of a particular peak in each model. For instance, a delta peak was present in models 9 to 16. The lower most plot indicates the model evidence, as estimated by the free‐energy, for each of the 16 models. (b) Log Bayes factors for the presence of each peak. These are obtained by averaging the free‐energy over models that feature a particular peak, and subtracting the average free‐energy over models without that peak. The dashed horizontal line indicates a log Bayes factor of 3, the commonly accepted threshold for ‘strong evidence’ (see Table 2). There is ‘decisive evidence’ for the presence of alpha and theta peaks, and ‘strong evidence’ for the presence of a beta peak. There is only weaker ‘positive evidence’ for the presence of a delta peak.

In the spectra for all subjects and in both conditions, a strong alpha peak is apparent, as well as possible delta, theta, and beta peaks. To assess the probability for the presence of these peaks, we invert the full model space for each of the subjects. After having estimated all models, we use Bayesian model comparison to identify the best model. Results of model comparison are shown in Figure 6. We report a winning model having all three peaks: delta, alpha, and beta, with decisive evidence for theta and alpha and strong evidence for beta. Model comparison also suggests positive—but not strong—evidence for the presence of a delta peak. In addition, delta peaks tends to be confounded with the high pass filtering effect applied to the aperiodic component (Gerster et al. 2022). For these two reasons, we decided to exclude the delta peak from our model.

#### 3.3.3 Effect of age

**FIGURE 7:**
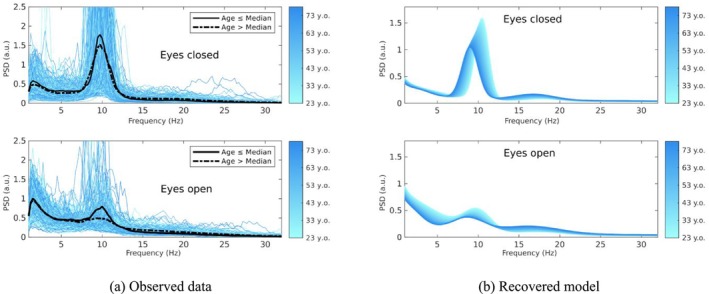
Observed spectrum and estimated model for the LEMON dataset. (a) Sample spectra in both eyes closed and eyes opened conditions for the 202 subjects. The plain black line shows the spectral average for subjects with a continuous regressor value below the population median, and the dashed line corresponds to the average for subjects above the median. Note that median split is only shown for visualisation purposes. The colour gradient indicates the age, with light cyan indicating 22.5 years old to dark blue indicating 77.5 years old. (b) Ribbon plot of the generative model, that is, the model recovered from the observed data in both rest and task conditions. The colour gradient indicates the age.

After identifying the optimal model, we construct a linear model that links the model parameters to subject age, like our simulated example. As we only have age ranges rather than exact ages, we approximate the subject's age using the mean of each age range. Parameter estimation at the group level is achieved using PEB. In Figure 7, we display sample spectra for all subjects, along with averaged spectra post‐median split by age. Additionally, we depict the interpolated group‐level spectral model across the entire age spectrum. While the ageing effect on the spectra from the median split is minimal, BSD effectively captures a prominent ageing effect on the overall spectrum. This exhibits a limitation of the median split approach (among other issues, see MacCallum et al. 2002), which can obscure the underlying effects of interest. The framework presented here enables the statistical evidence for different characterizations of between‐subjects effects to be specified and compared. For example, one could compare the evidence for a model in which a medium split is employed, thereby creating a categorical (dummy) variable in the between‐subjects design matrix, versus a model with a continuous regressor for age.

**FIGURE 8:**
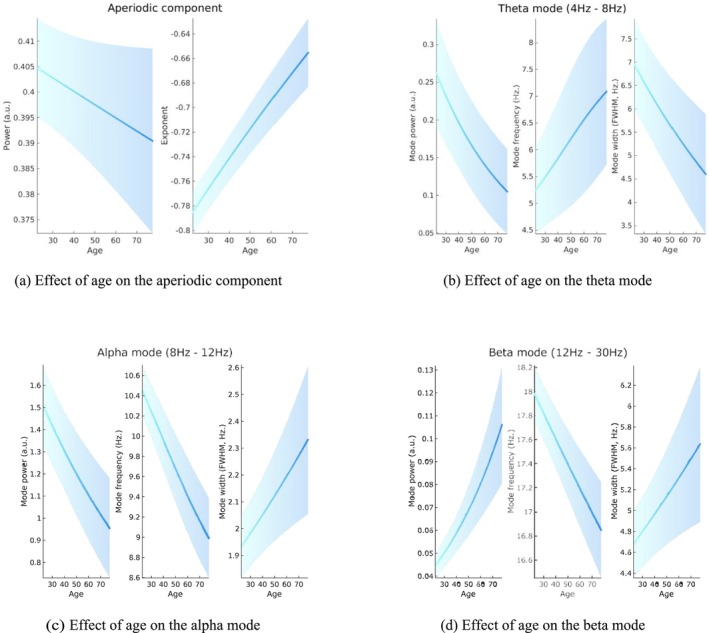
Effect of age on model parameters of the aperiodic component (a) and theta (b), alpha (c), and beta modes (d). For each plot, the blue line indicates regressed mean of the effect, whilst the shaded area surrounding the mean indicates the 90% credible interval derived from the posterior parameter variance.

Results for individual components are shown in Figure 8. We see that the power of the aperiodic component exhibits a slight decrease with age, and the slope of the aperiodic component flattens with age (Figure 8a). We report a decrease of theta frequency, with an increase of the power and a decrease of the width (Figure 8b). As expected, we find a strong decrease of the alpha peak power and frequency with age, as well as an increase of the peak width (Figure 8c). Surprisingly, we report an increase of the power of the beta peak with age, together with a decrease in frequency and an increase in width (Figure 8d). We report no effect of age on the between‐condition effects.

Most of these finding are in line with the existing ageing literature: age is related to an overall slowing of brain waves and a decrease of the slope of the aperiodic component (Tröndle et al. 2023; Merkin et al. 2023; Hill et al. 2022; Thuwal et al. 2021). Here, BSD allows us to quantify each slope, as well as their credible intervals, in a simple but rigorous manner.

## 4 Discussion

Our study introduces BSD, a novel method for analysing neural spectral power. Through a series of simulations and application to real EEG data, we have demonstrated its utility in identifying spectral peaks, selecting appropriate models, and investigating relationships between spectral parameters and continuous covariates such as age. Here, we discuss the implications of our findings and the potential future directions for research in this area.

Our results from the simulation study showcase the efficacy of BSD in model selection and parameter estimation. By comparing different model configurations using Bayesian model comparison, we were able to accurately identify the presence of spectral peaks and select the most appropriate model for the data. Furthermore, our comparison with FOOOF highlights the strengths of BSD in detecting spectral peaks, particularly in scenarios with intermediate peak heights. The use of variational inference in BSD allows for a principled approach to model selection, balancing between model accuracy and complexity. This makes BSD a conservative method, which favours simpler models when the data provide insufficient support for the presence of very small peaks.

Our approach differs from the recent model selection technique proposed by (Wilson et al. 2024), which uses the Bayesian information criterion for model selection. As observed in earlier work on variational Bayesian methods, Akaike and Bayesian information criteria are approximations to the variational free‐energy (Equation 12) and do not perform as well for model comparison, in the presence of correlation among parameters (Penny 2012). In contrasts, BSD specifies a generative model for the data which allows one to evaluate the free‐energy directly. This is a more principled approach to model selection, as the free‐energy considers correlations among parameters and therefore provides a more accurate estimate of the model evidence.

One notable advantage of BSD is its ability to incorporate continuous covariates into the analysis, as demonstrated using both simulated and real data. Notably, our approach allows one to perform linear statistics for group studies, similar to the recent GLM‐Spectrum (Quinn et al. 2024) or the earlier convolution GLM approaches (Litvak et al. 2013), while operating in the parameter space of the model. By modelling the relationship between spectral peak parameters and covariates using PEB, we were able to quantify how neural spectral features vary continuously with age.

The principled approach to group‐level analysis using BSD could enable new studies of large datasets in clinical neuroscience, for instance to understand the effects of ageing and the development of dementia in relation to environmental factors (Scally et al. 2018). In addition, BSD could be leveraged to analyse experimentally driven spectral changes reflecting slow mechanisms such as adaptation and learning, in conjunction with existing methodologies for analysing multiscale time series (Medrano et al. 2024). Less trivially, BSD could be used to analyse resting state fMRI signals, providing a phenomenological complement to existing mechanistic approaches (Friston et al. 2014).

One promising direction for future research is the integration of BSD with Dynamic Causal Modelling (DCM) for a comprehensive analysis of neural spectra. DCM offers a mechanistic framework for understanding causal interactions between different brain regions and how they give rise to observed neural activity. We suggest that BSD can be first be used to answer *phenomenological* questions, in other words, summarise *how* data features are changing. Then, DCM can be used to answer *mechanistic* questions and understand *why* data features are changing, relating changes in spectral power and coherence to changes in effective connectivity. Together, BSD and DCM would allow one to automatically identify robust spectral features and to relate them to the underlying mechanisms at play.

## 5 Conclusion

In this work, we introduced BSD, a Bayesian framework for constructing, analysing, and comparing spectral models of neural power. We have shown that the fundamentals of BSD rest on well‐established Bayesian methods that allow one to rigorously compare hypotheses and perform group‐level statistics on spectral data. Our results on simulated data show that BSD yields robust yet sensible results, outperforming FOOOF in identifying spectral peaks. In addition, we have shown how BSD can be used straightforwardly to perform group‐analysis on large datasets.

In conclusion, BSD offers a powerful and flexible approach for analysing neural spectral power, with applications ranging from basic research on brain function to clinical studies of neurological and psychiatric disorders. By leveraging advanced Bayesian modelling techniques with expressive parametric models of neural spectra, BSD opens new avenues for investigating the complex dynamics of brain activity and their relationship to behaviour and cognition.

## Author Contributions

**Johan Medrano:** conceptualization, investigation, visualization, writing – original draft preparation. **Nicholas A. Alexander:** methodology, writing – review and editing. **Robert A. Seymour:** methodology, writing – review and editing. **Peter Zeidman:** supervision, writing – review and editing.

## Conflicts of Interest

The authors declare no conflicts of interest.

### Peer Review

The peer review history for this article is available at https://www.webofscience.com/api/gateway/wos/peer‐review/10.1111/ejn.70149.

## Supporting information


**Figure S1.** Parameter recovery plot for the simulated data. The x‐axis shows the true value of the parameter, and the y‐axis shows the estimated value of the parameter. The plain line indicates the identity line, where the estimated value equals the true value. The blue colour gradient for the alpha and beta peak indicates the peak amplitude. The axis for the frequency and FWHM is in units of Hertz.

**Figure S2.** AUC‐ROC, i.e., the detection score, as a function of the peak SNR in decibels for both BSD (in plain green) and FOOOF (in dashed orange). Each row shows a different level of output noise: 0 dB (top), *−*17 dB (middle), and *−*35 dB (bottom). Columns corresponds to the frequency band in which the peaks are located: delta (1–4 Hz), theta (4–8 Hz), alpha (8–12 Hz), beta (12–30 Hz), and gamma (30–100 Hz). For FOOOF, the default parameters of the official Python implementation (specparam) have been used.

## Data Availability

The LEMON dataset used in the worked example is openly available at https://fcon_1000.projects.nitrc.org/indi/retro/MPI_LEMON.html. BSD is made available as a toolbox as part of the SPM software package, available at https://github.com/spm/spm/blob/main/toolbox/BSD.
